# Thyroid cancer in Luxembourg: a national population-based data report (1983–1999)

**DOI:** 10.1186/1471-2407-6-102

**Published:** 2006-04-24

**Authors:** René Scheiden, Marc Keipes, Carlo Bock, Walter Dippel, Nelly Kieffer, Catherine Capesius

**Affiliations:** 1 Division of pathology, National Health Laboratory, Luxembourg; 2 Department of endocrinology, Clinique Ste Thérèse, Luxembourg; 3 Department of oncology, Clinique Ste Thérèse, Luxembourg; 4 Morphologic Tumour Registry, National Health Laboratory, Luxembourg

## Abstract

**Background:**

Twenty years after the nuclear accident in Chernobyl (Eastern Europe), there is still a controversial debate concerning a possible effect of the radioactive iodines, especially I-131, on the increase of thyroid carcinomas (TCs) in Western Europe. Time trends in incidence rates of TC in Luxembourg in comparison with other European countries and its descriptive epidemiology were investigated.

**Methods:**

The population-based data of the national Morphologic Tumour Registry collecting new thyroid cancers diagnosed between 1983 and 1999 at a nation-wide level in the central division of pathology were reviewed and focused on incidence rates of TC. Data from 1990 to 1999 were used to evaluate the distribution by gender, age, histological type, tumour size and the outcome.

**Results:**

Out of 310 new thyroid carcinomas diagnosed between 1990 and 1999, 304 differentiated carcinomas (A: 80% papillary; B: 14.5% follicular; C: 3.5% medullary) and 6 anaplastic/undifferentiated TCs (D: 2%) were evaluated. The M/F-ratio was 1:3.2, the mean age 48.3 years (range: 13–92). The overall age-standardized (world population) incidence rates over the two 5-year periods 1990–1994 and 1995–1999 increased from 7.4 per 100,000 to 10.1 per 100,000 in females, from 2.3 per 100,000 to 3.6 per 100,000 in males. Only 3 patients were children or adolescents (1%), the majority of the patients (50%) were between 45 and 69 years of age. The percentage of microcarcinomas (<1 cm) was A: 46.4%, (115/248); B: 13.3%, (6/45); C: 27.3%, (3/11). The unexpected increase of TCs in 1997 was mainly due to the rise in the number of microcarcinomas. The observed 5-year survival rates for both genders were A: 96.0+/-2%; B: 88.9%; C: 90.9%; D: 0%. Prognosis was good in younger patients, worse in males and elderly, and extremely poor for undifferentiated TCs.

**Conclusion:**

The increasing incidence rates of TC, especially of the papillary type, seem mainly due to a rise in diagnosed microcarcinomas due to some extent to a change in histologic criteria and to more efficient diagnostic tools. This rise appears to be independent of the number of surgical treatments, the immigration rate, and the Chernobyl fallout as the incidence of TC in children remained stable.

## Background

Since April 1986, the day of the nuclear accident in Chernobyl (Ukraine) leading to the exposure of large populations to radioactive iodines (particularly I-131) in many Eastern European countries and to a lesser degree in Central and Western Europe, there has been an increased interest of the health care authorities to collect epidemiological data on cancer incidence, especially in leukaemia and thyroid cancer (TC) [1,2].

The study aim was to examine time trends (1983–1997) in the thyroid cancer age-standardized (world population) incidence rates in the Grand-Duchy of Luxembourg (Western Europe), an iodine deficient goitre endemic area, and to analyse the distribution of TC (1990–1999) by age, gender, histological type, tumour size, immigration rate and outcome.

One goal was to present the data on frequency of thyroid cancers in a goitre endemic area over a ten-year period, 1990–1999, and to identify recent changes, if any. Our objective was to assess whether these changes could be artefactual due a) to incorrect or changing histological diagnoses, b) to more complete case reporting in the post-Chernobyl era or c) to increased surgical interventions in individuals with an enlarged, diffuse or nodular pattern like thyroid gland in the years following the nuclear accident.

## Methods

The design of the study was a retrospective chart review from 1 January 1983 to 31 December 1999. The descriptive epidemiologic data of thyroid cancer in a selected area, the Grand-Duchy of Luxembourg, were provided by the national Morphologic Tumour Registry (MTR), which is the population-based cancer registry at national level which collects data on about 95% of the precancerous and cancerous lesions verified by microscopic examinations. These thyroid cancer cases had been exclusively diagnosed and "double-read" in the central division of pathology by nine senior pathologists. Over the last decade, the 1990s, the population at risk rose from 384,400 to 435,700, an average of 1.3 % per year [3]. Patients of all nationalities, races or ethnic origins living in Luxembourg and treated for thyroid cancer by surgery were considered. Disease histology was limited to the four major histological types, including papillary, follicular, medullary and anaplastic/undifferentiated carcinomas and their variants, diagnosed according to the WHO-classification [4]. Tumours of the thyroid gland recorded as other carcinomas, metastatic or recurrent disease, malignant carcinoid, lymphomas or mesenchymal malignant tumours were excluded from the current review. Over the observation period from 1983 to 1999, a total of 482 autopsies were performed in Luxembourg. None of these necropsies showed a suspected or incidental thyroid cancer.

Data on 5,611 surgical specimens resected by 41 senior surgeons in the nine hospitals of the country during the period 1990 to 1999 were evaluated. 5,301 thyroidectomy specimens showed benign lesions (i.e. solitary adenomas, parenchymatous diffuse or nodous goiter, Hashimoto's thyroditis, Graves' disease). 310 specimens were associated with a suspected or incidental thyroid carcinoma and allowed an interpretation in relation to tumour size, the local and regional spread, the lymph node and residual tumour status (R). They were analysed with respect to the TNM-system (i.e.: T: extent of primary tumour; N: regional lymph node metastasis; M: distant metastasis; R: residual tumour), according to the guidelines of the Union Internationale Contre le Cancer (UICC) and the American Joint Committee on Cancer (AJCC) [5,6]. The R-classification of the TNM-system was performed by conventional methods. In our series the histological examination of the nearest resection margin and the organ capsule was mandatory. R0 indicates no residual tumour; R1 corresponds to microscopically and R2 to macroscopically residual tumour, while Rx did not allow a statement on residual tumour.

Beside the evaluation of the frequency of the TCs by the 17 'classic' 5-year age categories (0–4 years to 80 years and above), the changes in incidence of thyroid carcinomas in relation to four cohorts of patients grouped in youngest (<20 years of age), younger (20–44 years), middle aged (45–69 years) and elderly (70 years and above) patients were reviewed.

The age-standardized (world population) incidence rates of TCs, diagnosed in the Grand-Duchy of Luxembourg during the period 1983–1997, were compared with the data of other geographical European regions with similar population density and socio-economic characteristics, published by the WHO in "Cancer in Five Continents", volumes VI, VII, VIII [7-11].

The observed 3-, 5- and 10-year survival rates of patients with TC were measured from the time of histopathologic diagnosis and calculated by the actuarial method (life-table) with a 95% confidence interval (c.i.). To determine the sample size for calculating the 95% c.i., the formula n × p × (1-p) ≥ 9 (n = number of considered patients, p = survival rate) was used. As the mortality tables of the Luxembourgish population in the late 1990s were not yet available, the relative 5-year survival rate from the period 1990–1994 could only be analysed.

The statistical evaluations included the chi-square test (χ^2^) with a level of significance p < 0.05 and the life-table survival analysis. The age-standardized incidence rates were calculated by the direct method, the standard error of the age-standardized rates by the Poisson approximation [12].

The fact that data sets on TC from different time periods were used [i.e. age-standardized (world population) incidence rates over the period 1983–1997 and crude incidence rates, age distribution, tumour sizes, histological subtypes or observed survival rates from 1990 to 1999] did not give rise to potential biases.

The files of the tumour registry (MTR) were also reviewed to detect the percentage of other malignant primaries developed in patients after an earlier TC. In Luxembourg, there is neither a systematic screening programme for early detection of TC nor an adequate programme of iodine supplementation.

## Results

During the period, 1990–1999, the histological diagnoses of 317 consecutive thyroid cancers removed by surgery were reviewed. A total of 5,611 surgical specimens resected for benign or malignant disease of the thyroid gland concerning 1,114 males and 4,497 females (M/F-ratio: 1:4) were examined. Seven malignancies in the thyroid gland were excluded (3 sarcomas, 2 Non-Hodgkin-lymphomas, 1 malignant plasmocytoma and 1 metastasis of a clear cell renal carcinoma). 111 of the 310 patients with TC were treated by lobectomy of the right thyroid lobe and 83 of the left lobe, 106 patients had total thyroidectomy. For 10 patients there was no precise clinical site description. The 310 thyroid carcinomas included 248 (80.0%) papillary, 45 (14.5%) follicular, 11 (3.5%) medullary and 6 (2.0%) anaplastic/undifferentiated carcinomas. The 310 carcinomas of the thyroid gland involved 74 males (23.9%) and 236 females (76.1%); M/F-ratio of 1:3.2. The mean age was 48.3 years (range: 13–92). 71 TCs (22.9%) had a multifocal growth pattern. 140 TCs were localized in the right thyroid lobe, 106 TCs in the left lobe and 54 TCs on both sides.

In the follow-up 11.6% of the 310 patients with primary TC developed a second primary malignant neoplasm in other organs. 33 out of the 236 female patients with TC had a subsequent second primary malignant neoplasm, 16 (7.1%) of these were breast cancers, whereas only 3 males out of 74 patients with TC suffered from a second primary carcinoma (larynx, kidney, urinary bladder).

The average, annual, age-specific rate of thyroid carcinomas in Luxembourg over the period 1990–1999 for all ages and both genders was 7.4 per 100,000: for males 3.6 per 100,000 and for females 11.3 per 100,000. The comparison of the crude incidence rates of the patients with TC diagnosed in the two 5-year periods 1990–1994 and 1995–1999 revealed an increase of 36.5% for both genders combined from 6.3 to 8.6 per 100,000, for females by 32.0% from 9.7 to 12.8 per 100,000 and for males by 51.7% from 2.9 to 4.4 per 100,000. Comparing the three 3-year time periods from 1990 to 1998 the overall crude incidence rates of TC in the two first periods, 1990–1992 and 1993–1995, remained stable at 6.3 per 100,000 and 6.0 per 100,000 respectively. In the third period, 1996–1998, there was a significant increase of the crude incidence rate of TC by 53.3%, from 6.0 per 100,000 to 9.2 per 100,000 (p < 0.05). Additional file 1 displays the crude incidence rates of TCs (n = 310 cases) for both genders in relation to the histological subtypes and the tumour sizes over the study period.

**Figure 1:**
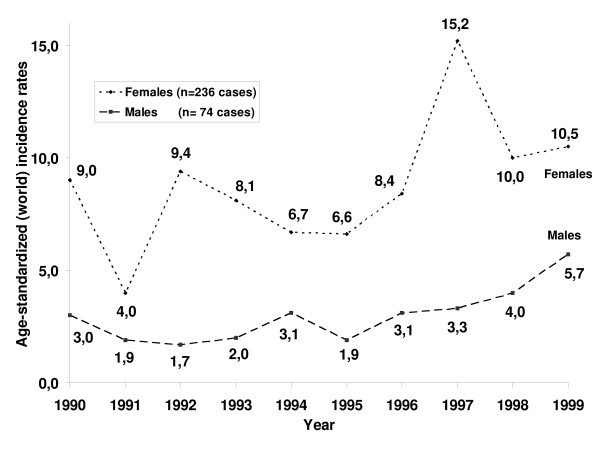
Time trend of the age-standardized (world population) incidence rates of thyroid carcinomas (n = 310) by gender; 1990–1999.

The average, annual, age-standardized incidence rate (ASR-world population) of thyroid carcinomas during the study period 1990–1999 was 5.9 per 100,000 for both genders: for males 3.0 per 100,000 and for females 8.8 per 100,000 (Figure 1). In Additional file 2 the world age-standardized incidence rates (ASR/W) of TCs in the Luxembourgish population (females and males) over the period 1983–1997 in comparison to other Western European countries, the USA and Canada are presented [7-11].

The total number of TCs rose from 31 cases in 1990 to 45 in 1999 (in females from 24 to 31, in males from 7 to 14). Comparing the two 5-year periods 1990–1994 to 1995–1999, there was a significant increase in the absolute number of all TCs from 126 to 184 cases (p < 0.001). The absolute number of TCs increased significantly in females over the two periods from 98 to 138 cases (p < 0.01), in males from 28 to 46 cases (p < 0.001). In 1997, there was an abnormal two-fold increase of the number of morphologically confirmed TCs especially in middle-aged females, which required further investigations focusing on tumour sizes and the number of surgical treatments.

**Figure 2:**
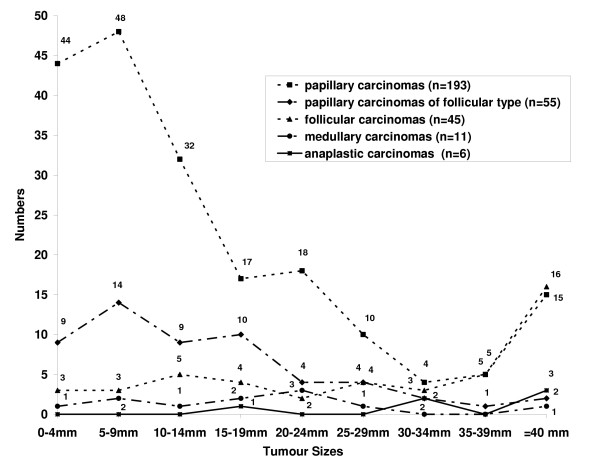
Number of TCs stratified by histological type and tumour size; n = 310 cases; period 1990–1999.

In our series 124 microcarcinomas – i.e. occult, small TCs with less than 1 cm in diameter – could be identified. 92 of these were of papillary type, 23 of papillary type variant follicular, 6 of follicular type and 3 of medullary type (Figure 2). Time trends of variations in tumour sizes (millimetres) are shown in Additional file 1. Indeed, in 1997 out of 49 TCs, 26 cases (53.1%) were microcarcinomas. This means a statistically significant 2.4-fold increase, in comparison to the average number of microcarcinomas diagnosed over the study period.

**Figure 3:**
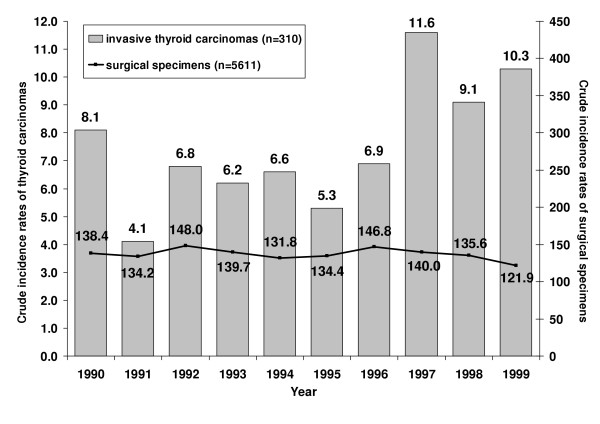
Crude incidence rates of invasive thyroid carcinomas and the surgical specimens for benign or malignant diseases for both genders, period: 1990–1999.

During the same time (1990–1999) the number of surgical specimens examined (n = 5,611 cases) remained stable, 532 specimens in 1990 and 531 in 1999. In Figure 3 the annual crude incidence rates of TC and the annual crude incidence rates of the surgical specimens are represented.

**Figure 4:**
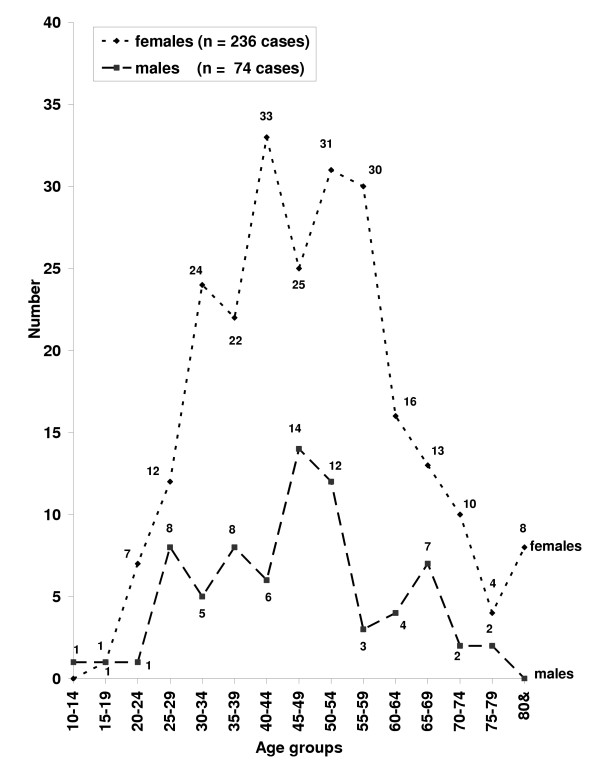
Age-distribution of the thyroid carcinomas (n = 310 cases); 1990–1999.

The age-distribution of the 310 patients with TC is documented in Figure 4. In our series only 3 patients (1%) were children or adolescents, whereas 126 patients (40.6%; 98 females/28 males) were between 20 and 44 years old, 155 patients (50.0%; 115 females/40 males) between 45 and 69 years and 26 patients (8.4%; 22 females/4 males) 70 years of age and above.

The overall observed survival rates of the 310 patients with TC, diagnosed on surgical resection specimens between 1990 and 1999, were calculated and stratified by years: 1-year: 95.2+/-2% (n = 295/310); 2-year: 95.2+/-2% (n = 295/310); 3-year: 93.9+/-3% (n = 291/310); 4-year: 93.2+/-3% (n = 289/310) and 5-year:92.9+/-3% (n = 288/310). The overall observed 3-year survival rate of the 310 patients with TC was 93.9+/-3% (291/310) and the 5-year observed survival rate 92.9+/-3% (288/310).

Over the first study period, 1990–1994, the relative 3-year survival rate of the 126 patients with TC was 93.9+/-4% for both genders (females: 95.5+/-4%; males: 88.2%), the relative 5-year survival rate 94.2+/-4% for both genders (females 95.4+/-4%; males: 90.1%).

In Table 1 the observed 3-, 5- and 10- year survival rates of TC for both genders in relation to the four histopathological types – papillary, follicular, medullary and anaplastic/undifferentiated carcinomas – and the four cohorts <20 years, 20–44 years, 45–69 years and >69 years of age are documented. All patients with differentiated TC aged 44 years or less had good prognosis and all patients survived 5 years. Additional file 3 shows the observed 3-, 5- and 10-year survival rates of TC by time periods, gender and histological type.

In Table 2, the observed 5-year survival rate of 253 patients with TC is stratified by tumour size (pT/UICC) and by surgical resection for cure (R0). 14 patients with R1- and R2-status after the first thyroid gland lobectomy got at least one week later total thyroidectomy for cure. 52 patients with TC were classified as Rx-cases because the thyroid gland was removed in several irregular fragments.

All 6 patients with an anaplastic/undifferentiated TC died within the first 3 months after the histopathologic diagnosis (survival range: 5–86 days).

## Discussion

Thyroid cancer incidence rates have increased steadily in Luxembourg and elsewhere. Over the observation period, 1990–1999, the overall crude incidence rates of thyroid carcinomas in Luxembourg rose from 6.4 per 100,000 (1990–1994) to 8.6 per 100,000 (1995–1999). A similar increase of the age-standardized (world population) incidence rates of TC during the three 5-year periods (1983–87; 1988–92; 1993–97) has also been reported in many other Western European and overseas countries (Additional file 2) [2,9-11,13-16]. In our series mainly women of childbearing age (60%) were at highest risk. At the same time 7.1% of our female patients with TC developed a second primary breast cancer. Some authors believe that sex hormones may play a role in the etiology of these tumours [17,18]. An early exposure to common risk factors or genetic susceptibility of certain subpopulations for both malignancies may be plausible [19]. Adjadj et al. investigated, in a series of 2,365 women who were treated for thyroid carcinoma, 48 women (2.0%) with a subsequent breast carcinoma. They did not find a relationship with I-131 treatment and/or external radiotherapy [20].

It is generally accepted that the thyroid gland is highly sensitive to radiation-induced oncogenesis [1,15,18,21-23]. It has been demonstrated that exposure to radiation at moderate or high levels can cause most forms of cancer. Leukaemia and cancers of the breast, lung and thyroid are particularly sensitive to induction by radiation, especially at young ages at exposure (i.e. atmospheric nuclear weapon programmes in the US and former Soviet Union, atomic bombing in Japan in 1945) [24]. Substantial evidence is also derived from groups exposed for environmental reasons, radiotherapeutically or occupationally, i.e. underground hard rock miners who inhaled radioactive radon gas and its decay products, patients irradiated therapeutically for head and neck tumours, medical staff members. The degree of carcinogenic risk arising from low levels of radiation exposure is more contentious, but the available evidence points to an increased risk that is approximately proportional to the dose received [24,25]. In the US radiologic technologists health study from 1983 to 1998 analysing the cancer incidence in medical staff exposed to low doses of radiation, an elevated risk for thyroid cancer in both genders was reported. The observed excesses in this cohort may also reflect, at least in part, an earlier detection among medical staff with easy access to health care procedures [26].

The thyroid gland of children is particularly vulnerable to the carcinogenic action of ionizing radiation. This may explain why the incidence of TC in children in the Chernobyl area increased to a peak exceeding 10 per 100,000 in this area after the nuclear accident [1,18,21]. Additionally it has been reported that probably at least some of the children, who are treated by external fractionated radiotherapy for cancer, are predisposed to develop a later TC [25,27]. In contrast to some Eastern European countries, such as Belarus, Ukraine, Poland, or even Great Britain, we did not find an increase of childhood thyroid cancers (Figure 3) [1,22,28]. Only 3 cases of TC in children and adolescents of 20 years of age or less, could be diagnosed in our series of the 1990s. This does not support the hypothesis of increased radiation-associated TC in Luxembourg following the exposure to nuclear fallout after the Chernobyl accident as a causative effect. This is consistent with the low individual (mean) dose of radiation following the accident 20 years ago which the Luxembourgish population was exposed to. The dose measured from 1 May 1986 to 1 May 1987 by the radioprotection division staff of the National Health Directorate (Ministry of Health) and which did not vary across the country, was 12.2 millirem. During the same period, the radioactive exposure in the Netherlands was 6.1 millirem and 21 millirem in Switzerland [29]. Similar observations in France were reported by Verger P et al. [2].

In our study, the large majority of TC (80%) was of the papillary histopathological subtype, 14.5% were follicular, 3.5% medullary and 2.0% anaplastic/undifferentiated carcinomas. Bhattacharyya et al. published 18,118 cases of differentiated thyroid carcinoma cases from the US Surveillance, Epidemiology and End-Results (SEER) cancer registry programme database, registered between 1988 and 1998 [30]. These identified cases included 87.3% papillary, 9.9% follicular and 2.8% medullary carcinomas. The differences in the percentage of papillary and follicular carcinomas may be attributable to a more strict application of the histopathologic WHO-classification in our department. Indeed, since 1995, we used in particular the diagnosis of the papillary TC, variant follicular (i.e. Lindsay tumour) in 55 cases (Figure 2). In the early 1990s these tumours with so called ground-glass nuclei were sometimes classified as atypical follicular adenomas when they were encapsulated or as follicular TC when a capsular and vascular infiltration could be detected. This changing diagnostic practice may partially explain the particularly low percentage of follicular TC in our series in the second study period. A similar shift in histopathological diagnoses from follicular to papillary TC has been observed in Switzerland by Verkooijen et al. [31].

It has been argued that many of the reported TC cases could correspond to a dormant and thus clinically irrelevant type of carcinoma, a so-called occult TC [31-34]. These silent carcinomas were often considered as non-palpable 'microcarcinomas' of less than 1 centimetre of size. In fact, a large and noticeable proportion of our 310 TCs were differentiated microcarcinomas including 46.4% (115/248) papillary TCs, 13.3% (6/45) follicular and 27.3% (3/11) medullary thyroid carcinomas (Additional file 1, Figure 2). In the literature a comparable increase of the percentage of microcarcinomas of TCs over the last 30 years, varying between 7% and 16.6%, has been reported [31,35].

To explain the two-fold increase of the diagnosed TC in 1997 in our series, we reviewed the patient data in our files and analysed the findings in relation to the patients' nationalities and the tumour sizes. Luxembourg is a country with a high immigration rate, 29.4% of the residents came from abroad (census data 1991, STATEC). Because only 10 of our 49 patients with TC (20.4%) diagnosed in 1997 were from abroad, a relationship between the TC increase and the immigration rate is not likely. The analysis of the tumour sizes of TC showed that in 1997, there was also a two-fold increase of the incidental microcarcinomas of the papillary type, which may be the major cause of the peak in 1997. The number of surgical treatments for enlarged thyroid gland decreased slightly. If we consider the crude incidence rates for both, TC and surgical specimens, the findings remained stable over the study period (Figure 3). Because there is no relationship between the frequency of microcarcinomas and the absolute number of surgical treatments, it may be that improved image giving procedures (i.e. ultrasound, computer scanner) play a role in the detection of more suspicious nodular lesions of less than 1 centimetre in greatest size [36]. The same holds true for a study of 471 cases from France between 1980 and 2000. There was a rise in prevalence of TCs among operated patients from 12% to 37%, although the proportion of patients undergoing surgery remained stable [16].

The high percentage of patients with thyroid microcarcinomas (<1 cm), particularly of the differentiated papillary type, may also be one reason for the high survival rates in our series (Table 2). Thyroid carcinomas generally have good prognosis, especially in patients of 44 years of age or less, except for undifferentiated carcinomas (none of our 6 patients survived three months). Long-term follow-up beyond 10 or 20 years is needed to clearly demonstrate an excess in mortality as a consequence of the increasing number of diagnosed TCs. The observed 3-, 5- and 10-year survival rates (Additional file 1) in relation to the different histopathological tumour types and the genders demonstrated a better prognosis in both genders over the two time periods (1990–1994 and 1995–1999) and decreasing survival rates for papillary TC, followed by follicular, medullar and anaplastic carcinomas. The prognosis in general was worse in men than in women. We found 10-year survival rates between 71.4% and 90.4% (Additional file 3).These data are in concordance with the findings of others reporting independent of the histological type, an overall 10-year survival rate varying between 74.0% and 89.4% [13,21,35,37,38]. The relative survival rates for TC patients would give better data for TC in Luxembourg than indicated by the observed survival rates reported here.

## Conclusion

The increasing thyroid cancer incidence rates especially of the papillary subtype seem mainly due to the increase in the diagnosed microcarcinomas over the last 5 years due to some extent to changes in histological diagnostic criteria (i.e. atypical follicular adenoma with so-called ground-glass nuclei being classified now as papillary TC follicular variant). Particularly female patients at childbearing age were concerned. The number of thyroid surgery over the study period remaining stable, the increase of incidentally discovered TC cases may also be linked to the use of more efficient diagnostic tools. Changes in immigration rates had no influence on the number of diagnosed TCs and neither did the Chernobyl fallout since no rise in the number of childhood cancers was found. Survival rates in males and the elderly were worse than in women and younger patients. The prognosis of undifferentiated thyroid cancers was extremely poor.

## Competing interests

The author(s) declare that they have no competing interests.

## Authors' contributions

All authors (RS, MK, CB, WD, NK, CC) collaborated intensely on all aspects of the manuscript, from research design to data presentation. RS and CB wrote the article. MK, WD and NK helped to draft the manuscript. RS and CC managed the data collection and data verifications. RS performed the statistical analysis. All authors approved the final manuscript.

## Pre-publication history

The pre-publication history for this paper can be accessed here:



## Supplementary Material

Additional file 1 Crude incidence rates of the thyroid carcinomas (n = 310 cases) by histological type and tumour sizes, both genders; period 1990–1999. Click here for file

Additional file 2 Thyroid gland cancer in the European Community: world age-standardized incidence rates [ASR(W)***], time trends: 1983–1987; 1988–1992; 993–1997. Click here for file

Additional file 3 Prognoses of thyroid carcinomas removed by surgery stratified by histological type and both genders; period 1990–1999. Click here for file

## Figures and Tables

**Table 1:** Overall observed 3-, 5- and 10-year survival rates of TCs for both genders in relation to the 4 age cohorts (<20 years, 20–44 years, 45–69 years and >69 years of age) and the 4 histological types (papillary, follicular, medullary and anaplastic carcinomas); n = 310 cases; period 1990–1999.

**1990–1994**												
	**Papillary carcinomas**			**Follicular carcinomas**			**Medullary carcinomas**			**Anaplastic carcinomas**		

	3 y	5 y	10 y	3 y	5 y	10 y	3 y	5 y	10 y	3 y	5 y	10 y

**<20 years**	(0/0)	(0/0)	(0/0)	(0/0)	(0/0)	(0/0)	(0/0)	(0/0)	(0/0)	(0/0)	(0/0)	(0/0)
**20–44 y.**	**100% **(42/42)	**100% **(42/42)	97.6%* (41/42)	**100% **(9/9)	**100% **(9/9)	88.9%* (8/9)	(0/0)	(0/0)	(0/0)	(0/0)	(0/0)	(0/0)
**45–69 y.**	91.8%* (45/49)	89.8%* (44/49)	87.8%* (43/49)	90.9%* (10/11)	90.9%* (10/11)	81.8%* (9/11)	66.7%* (2/3)	66.7%* (2/3)	66.7%* (2/3)	(0/0)	(0/0)	(0/0)
**>69 years**	66.7%* (2/3)	33.3%* (1/3)	**0% **(0/3)	57.1%* (4/7)	42.9%* (3/7)	42.9%* (3/7)	**100% **(1/1)	**100% **1/1)	**100% **(1/1)	**0% **(0/1)	**0% **(0/1)	**0% **(0/1)

**1995–1999**												

	**Papillary carcinomas**			**Follicular carcinomas**			**medullary carcinomas**			**Anaplastic carcinomas**		

	3 y	5 y	10 y**	3 y	5 y	10 y**	3 y	5 y	10 y**	3 y	5 y	10 y**

**<20 years**	**100% **(3/3)	**100% **(3/3)	-	(0/0)	(0/0)	-	(0/0)	(0/0)	-	(0/0)	(0/0)	-
**20–44 y.**	**100% **(65/65)	**100% **(65/65)	-	**100% **(7/7)	**100% **(7/7)	-	**100% **(3/3)	**100% **(3/3)	-	(0/0)	(0/0)	-
**45–69 y.**	98.7%* (76/77)	98.7%* (76/77)	-	**100% **(9/9)	**100% **(9/9)	-	**100% **(4/4)	**100% **(4/4)	-	**0% **(0/2)	**0% **(0/2)	-
**>69 years**	88.9%* (8/9)	77.8%* (7/9)	-	50%* (1/2)	50%* (1/2)	-	(0/0)	(0/0)	-	**0% **(0/3)	**0% **(0/3)	-

**1990–1999**												

	**Papillary carcinomas**			**Follicular carcinomas**			**medullary carcinomas**			**Anaplastic carcinomas**		

	3 y	5 y	10 y**	3 y	5 y	10 y**	3 y	5 y	10 y**	3 y	5 y	10 y**

**<20 years**	**100% **(3/3)	**100% **(3/3)	-	(0/0)	(0/0)	-	(0/0)	(0/0)	-	(0/0)	(0/0)	-
**20–44 y.**	**100% **(107/107)	**100% **(107/107)	-	**100% **(16/16)	**100% **(16/16)	-	**100% **(3/3)	**100% **(3/3)	-	(0/0)	(0/0)	-
**45–69 y.**	96.0%* (121/126)	95.2%* (120/126)	-	95.0%* (19/20)	95%* (19/20)	-	85.7%* (6/7)	85.7%* (6/7)	-	**0% **(0/2)	**0% **(0/2)	-
**>69 years**	83.3%* (10/12)	66.7%* (8/12)	-	55.6%* (5/9)	44.4%* (4/9)	-	**100% **(1/1)	**100**% (1/1)	-	**0% **(0/4)	**0% **(0/4)	-

* sample size too small for calculating the 95% confidence interval

** data not yet available

**Table 2:** Prognoses of differentiated thyroid carcinomas resected for cure (R0)- overall observed 5-year survival rates 1990–1999 stratified by tumour size (253 cases; exclude 6 anaplastic carcinomas).

**Observed 5-year survival rates R0**			
**pT1 (0.1–0.9 cm)**	n = 172 cases	97.1%*	(168/172)

0.1–0.4 cm	n = 52 cases	98.1%*	(51/52)
0.5–0.9 cm	n = 57 cases	100%	(57/57)
1.0–1.4 cm	n = 38 cases	97.4%*	(37/38)
1.5–1.9 cm	n = 25 cases	92.0%*	(23/25)

**pT2 (2.0–3.9 cm)**	n = 44 cases	97.7%*	(43/44)

2.0–2.9 cm	n = 30 cases	100%	(30/30)
3.0–3.9 cm	n = 14 cases	92.9%*	(13/14)

**pT3 (= 4 cm)**	n = 21 cases	85.7%*	(18/21)


**pT4 (any size, extend beyond thyroid capsule)**	n = 16 cases	93.8%*	(15/16)

* sample size too small for calculating the 95% confidence interval
