# Supplementary material for: Thyroid cancer in Luxembourg: a national population-based data report (1983–1999)
Source: BMC Cancer. 2006 Apr 24;6:102. doi: 10.1186/1471-2407-6-102 (PMC1475873; doi:10.1186/1471-2407-6-102)
Supplement: Additional file 1 — Crude incidence rates of the thyroid carcinomas (n = 310 cases) by histological type and tumour sizes, both genders; period 1990–1999. [file 1471-2407-6-102-S1.doc]

|  | **papillary carcinoma (n=248)** | **follicular carcinomas (n=45)** | **medullary carcinomas (n=11)** | **anaplastic carcinomas (n=6)** | **Total**  **(n=310)** | **0-4 mm**  **(n=57)** | **5-9 mm**  **(n=67)** | **10-19 mm**  **(n=81)** | **20-29 mm**  **(n=46)** | **30-39 mm**  **(n=22)** | **>39 mm**  **(n=37)** |
| --- | --- | --- | --- | --- | --- | --- | --- | --- | --- | --- | --- |
| **1990** | 4.9 (19) | 2.9 (11) | 0.3 (1) | 0.0 (0) | 8.1 (31) | 1.6 (6) | 0.8 (3) | 2.1 (8) | 0.3 (1) | 1.3 (5) | 2.1 (8) |
| **1991** | 3.1 (12) | 1.0 (4) | 0.0 (0) | 0.0 (0) | 4.1 (16) | 0.5 (2) | 0.5 (2) | 1.0 (4) | 1.0 (4) | 0.0 (0) | 1.0 (4) |
| **1992** | 5.3 (21) | 1.3 (5) | 0.3 (1) | 0.0 (0) | 6.8 (27) | 0.8 (3) | 1.3 (5) | 3.3 (13) | 0.5 (2) | 0.3 (1) | 0.8 (3) |
| **1993** | 5.7 (23) | 0.0 (0) | 0.2 (1) | 0.2 (1) | 6.2 (25) | 1.0 (4) | 1.7 (7) | 1.7 (7) | 0.7 (3) | 0.2 (1) | 0.7 (3) |
| **1994** | 4.7 (19) | 1.7 (7) | 0.2 (1) | 0.0 (0) | 6.6 (27) | 0.5 (2) | 1.7 (7) | 2.0 (8) | 0.7 (3) | 0.7 (3) | 1.0 (4) |
| **1995** | 4.6 (19) | 0.2 (1) | 0.0 (0) | 0.5 (2) | 5.3 (22) | 0.5 (2) | 0.7 (3) | 1.7 (7) | 1.9 (8) | 0.2 (1) | 0.2 (1) |
| **1996** | 5.5 (23) | 1.2 (5) | 0.0 (0) | 0.2 (1) | 6.9 (29) | 1.7 (7) | 1.4 (6) | 1.2 (5) | 0.7 (3) | 1.0 (4) | 1.0 (4) |
| **1997** | 10.1 (43) | 0.9 (4) | 0.5 (2) | 0.0 (0) | 11.6 (49) | 3.1 (13) | 3.1 (13) | 3.1 (13) | 1.7 (7) | 0.5 (2) | 0.2 (1) |
| **1998** | 7.2 (31) | 1.2 (5) | 0.5 (2) | 0.2 (1) | 9.1 (39) | 2.1 (9) | 1.9 (8) | 1.9 (8) | 1.6 (7) | 0.7 (3) | 0.9 (4) |
| **1999** | 8.7 (38) | 0.7 (3) | 0.7 (3) | 0.2 (1) | 10.3 (45) | 2.1 (9) | 3.0 (13) | 1.8 (8) | 1.8 (8) | 0.5 (2) | 1.1 (5) |

Additional file 1. Crude incidence rates of the thyroid carcinomas (n=310 cases) by histological type and tumour sizes, both genders; period

1990-1999
