# Supplementary material for: Thyroid cancer in Luxembourg: a national population-based data report (1983–1999)
Source: BMC Cancer. 2006 Apr 24;6:102. doi: 10.1186/1471-2407-6-102 (PMC1475873; doi:10.1186/1471-2407-6-102)
Supplement: Additional file 2 — Thyroid gland cancer in the European Community: world age-standardized incidence rates [ASR(W)***], time trends: 1983–1987; 1988–1992; 993–1997. [file 1471-2407-6-102-S2.doc]

| Males | **83-87** | **88-92** | **93-97** |  | **Females** | **83-87** | **88-92** | **93-97** |  |
| --- | --- | --- | --- | --- | --- | --- | --- | --- | --- |
|  |  |  |  |  |  |  |  |  |  |
| Spain / Navarra | 0.6 | 2.2 | 2.6 | ↑ | **Luxembourg*** | **3.0** | **6.7** | **8.8** | ↑ |
| Italy / Parma | 1.6 | 2.2 | 2.5 | ↑ | Spain / Navarra | 4.9 | 6.9 | 7.9 | ↑ |
| **Luxembourg*** | **1.5** | **2.0** | **2.5** | ↑ | Finland | 5.8 | 6.4 | 7.8 | ↑ |
| Finland | 1.8 | 1.8 | 2.3 | ↑ | Switzerland / St.Gall | 4.2 | 4.5 | 6.2 | ↑ |
| Switzerland / St.Gall | 2.4 | 1.6 | 2.1 | ↓ | Italy / Parma | 5.5 | 3.5 | 5.4 | ↓ |
| Germany / Saarland | 1.8 | 2.0 | 2.1 | ↑ | France / Bas Rhin | 2.9 | 2.6 | 4.8 | ↑ |
| France / Bas Rhin | 1.5 | 1.3 | 1.9 | ↑ | Germany / Saarland | 3.6 | 3.5 | 4.8 | ↑ |
| Netherlands / Maastricht | 0.7 | 1.0 | 1.6 | ↑ | Sweden | 3.8 | 3.6 | 3.5 | ↓ |
| Sweden | 1.6 | 1.4 | 1.3 | ↓ | Denmark | 2.0 | 2.1 | 2.3 | ↑ |
| Denmark | 1.0 | 0.8 | 1.0 | ↔ | Netherlands / Maastricht | 2.2 | 1.9 | 2.1 | ↓ |
| United Kingdom / South Western | 1.0 | 0.7 | 0.9 | ↓ | United Kingdom / South Western | 1.7 | 2.0 | 2.1 | ↑ |
| Ireland / Southern | 1.0 | 0.5 | 0.9 | ↓ | Ireland / Southern | 2.2 | 2.5 | 1.9 | ↓ |
|  |  |  |  |  |  |  |  |  |  |
| Canada | 1.7 | 1.8 | 2.1 | ↑ | Canada | 4.3 | 4.9 | 6.4 | ↑ |
| USA/Seer**, white | 2.2 | 2.5 | 2.8 | ↑ | USA/Seer**, white | 5.8 | 6.4 | 7.7 | ↑ |

* Monographies-New Cancer cases in Luxembourg [http://www.cancer-registry.lu] [7, 8]

** SEER: Surveillance, Epidemiology and End Results Program

*** Cancer incidence in five continents. Vol VI; Vol VII; Vol VIII [9, 10, 11]

Additional file 2. Thyroid gland cancer in the European Community: world age-standardized incidence rates [ASR(W)***], time trends: 1983-1987; 1988-1992; 1993-1997.
