# Supplementary material for: Thyroid cancer in Luxembourg: a national population-based data report (1983–1999)
Source: BMC Cancer. 2006 Apr 24;6:102. doi: 10.1186/1471-2407-6-102 (PMC1475873; doi:10.1186/1471-2407-6-102)
Supplement: Additional file 3 — Prognoses of thyroid carcinomas removed by surgery stratified by histological type and both genders; period 1990–1999. [file 1471-2407-6-102-S3.doc]

|  | **Observed survival rates** (actuarial method, 95% confidence interval) | | | | | | | | | | | |
| --- | --- | --- | --- | --- | --- | --- | --- | --- | --- | --- | --- | --- |
|  | **3 years** | | | | **5 years** | | | | **10 years** | | | |
|  | FEMALES | | MALES | | FEMALES | | MALES | | FEMALES | | MALES | |
| **Papillary carcinomas:** | | |  |  |  |  |  |  |  |  |  |  |
| 1990-1994 | **n=73** | 95.9%* | **n=21** | 90.5%* | **n=73** | 93.2%* | **n=21** | 90.5%* | **n=73** | 90.4%* | **n=21** | 85.7%* |
| 1995-1999 | **n=118** | 99.2%* | **n=36** | 97.2%* | **n=118** | 98.3%* | **n=36** | 97.2%* |  | **- **** |  | **- **** |
| 1990-1999 | **n=191** | 97.9%* | **n=57** | 94.7%* | **n=191** | 96.3%* | **n=57** | 94.7%* |  | **- **** |  | **- **** |
| **Follicular carcinomas:** | | |  |  |  |  |  |  |  |  |  |  |
| 1990-1994 | **n=20** | 90.0%* | **n=7** | 71.4%* | **n=20** | 90.0%* | **n=7** | 71.4%* | **n=20** | 80.0%* | **n=7** | 71.4%* |
| 1995-1999 | **n=11** | 100% | **n=7** | 85.7%* | **n=11** | 100% | **n=7** | 85.7%* |  | **- **** |  | **- **** |
| 1990-1999 | **n=31** | 93.5%* | **n=14** | 78.6%* | **n=31** | 93.5%* | **n=14** | 78.0%* |  | **- **** |  | **- **** |
| **Medullary carcinomas:** | |  |  |  |  |  |  |  |  |  |  |  |
| 1990-1994 | **n=4** | 75.0%* | **n=0** | / | **n=4** | 75.0%* | **n=0** | / | **n=4** | 75.0%* | **n=0** | / |
| 1995-1999 | **n=5** | 100% | **n=2** | 100% | **n=5** | 100% | **n=2** | 100% |  | **- **** |  | **- **** |
| 1990-1999 | **n=9** | 88.9%* | **n=2** | 100% | **n=9** | 88.9%* | **n=2** | 100% |  | **- **** |  | **- **** |
| **Anaplastic/undiff. carcinomas:** | | |  |  |  |  |  |  |  |  |  |  |
| 1990-1994 | **n=1** | 0% | **n=0** | / | **n=1** | 0% | **n=0** | / | **n=1** | 0% | **n=0** | / |
| 1995-1999 | **n=4** | 0% | **n=1** | 0% | **n=4** | 0% | **n=1** | 0% |  | **- **** |  | **- **** |
| 1990-1999 | **n=5** | 0% | **n=1** | 0% | **n=5** | 0% | **n=1** | 0% |  | **- **** |  | **- **** |
|  |  |  |  |  |  |  |  |  |  |  |  |  |
| * sample size too small for calculating the 95% confidence interval  ** data not yet available | | | | | | | | | | | | |

Additional file 3. Prognoses of thyroid carcinomas removed by surgery stratified by histological type and both genders; period 1990-1999.
